# Serum metabolomics reveals stage-dependent metabolic transitions during CIDR-PGF_2_α-induced estrus in Kazak ewes

**DOI:** 10.3389/fvets.2026.1904685

**Published:** 2026-07-31

**Authors:** Ullah Yaseen, Jinglei Wang, Mengqi Hou, Jia Kang, Mingshan Li, Xiang Wu, Yan Ruge, Zongsheng Zhao

**Affiliations:** College of Animal Science and Technology, Shihezi University, Shihezi, China

**Keywords:** acylcarnitine, CIDR-PGF_2_α, estrus synchronization, Kazak ewes, one-carbon metabolism, reproductive biomarkers, serum metabolomics, sphingolipid

## Abstract

**Background:**

Serum metabolomics is a powerful approach for characterizing the systemic biochemical changes accompanying reproductive transitions in livestock. A hormone-based protocol, CIDR-PGF_2_α, is used to drive Kazak ewes through a defined sequence of reproductive stages: the LP (luteal phase, early stage), LDP (luteolysis, middle stage), and OES (estrus, late stage when the ewe exhibits estrus). However, the serum metabolic dynamics during these transitions remain uncharacterized.

**Methods:**

Serum samples were collected from 18 Kazak ewes at the three stages LP, LDP, and OES (*n* = 6 per group) after they received CIDR-PGF_2_α synchronization treatment. Untargeted serum metabolomics was performed using a technique called UHPLC-MS/MS (Vanquish–Orbitrap Exploris 120). Differential metabolites were identified between stages using two statistical methods: OPLS-DA (VIP > 1.0) combined with Student's *t*-test (*p* < 0.05), and pathway enrichment analysis was conducted using the KEGG database for sheep (*Ovis aries)*.

**Results:**

A total of 1,861 metabolites were identified. Of these, 213, 119, and 203 were significantly different in the LP vs. LDP, LP vs. OES, and LDP vs. OES. All OPLS-DA models demonstrated strong ability to distinguish between stages (*R*^2^*Y* = 0.994, 0.989, and 0.995; *Q*^2^ = 0.580, 0.296, and 0.423), and 200-permutation testing confirmed the absence of overfitting. KEGG enrichment analysis identified 15 significantly enriched pathways in the LP vs. LDP, 11 in LP vs. OES, and 15 significantly enriched pathways in LDP vs. OES (*p* < 0.05). Notably, these pathways centered on four key areas of metabolism: (1) choline-mediated one-carbon metabolism, (2) mitochondrial fatty acid β-oxidation, (3) sphingolipid signaling, and (4) glycerophospholipid biosynthesis. Choline and betaine aldehyde dropped markedly during the middle stage LDP and partially recovered at the late stage OES. Two short-chain acylcarnitines, 3-hydroxybutyrylcarnitine and acetyl carnitine accumulated progressively from the early stage LP through to the late stage OES, reflecting increasing fatty acid β-oxidation activity. Sphingosine was the only metabolite that was consistently downregulated across all three comparisons (FC = 0.604, 0.336, and 0.556), a pattern consistent with sustained pro-survival sphingosine-1-phosphate signaling throughout the transition. Pregnanediol 3-O-glucuronide showed the largest fold change of any metabolite at OES (FC = 4.6), which verifies efficient hepatic progesterone clearance following luteolysis.

**Conclusion:**

In summary, this study provides the first serum metabolomics characterization of Kazak ewes undergoing CIDR-PGF_2_α-induced estrus. It identified five metabolites, choline, betaine aldehyde, 3-hydroxybutyrylcarnitine, acetyl carnitine, and sphingosine, as physiologically meaningful, stage-sensitive biomarkers of the reproductive transition. These results provide a quantitative metabolic baseline for Kazak ewe reproductive physiology and set the stage for future comparative metabolomics studies in naturally cycling ewes. The ultimate aim is to develop metabolite-targeted nutritional strategies to induce estrus during the non-breeding season.

## 1 Introduction

Sheep farming plays an important role in global livestock production, supplying meat, wool, and dairy products, and contributes greatly to the agricultural economy. Kazak sheep varieties, particularly in Xinjiang, China, are an important local genetic resource. Reproductive efficiency is one of the most important factors determining animal productivity, and improved estrus synchronization technology is a key strategy for improving sheep breeding management (1). Controlled internal drug release (CIDR) and the administration of Prostaglandin F_2α_ (PGF_2_α) are among the most widely accepted protocols for embryo synchronization as they reliably induce luteolysis and synchronize the estrous cycle, thus facilitating the artificial insemination (2, 3). The CIDR-PGF_2α_ protocol leads ewes through a series of reproductive stages, each with its own hormonal and physiological characteristics, the luteal phase (LP), the luteolysis phase (LDP), and the estrus phase (OES). Although the endocrine regulation of these stages has been well documented, it remains unclear how dynamic changes in serum metabolism occur in the LP, LDP, and OES phases of hormone-induced estrus.

Metabolomics is the comprehensive analysis of all small molecules or metabolites present in the biological system and represents the lowest level of biological information and directly reflects the functional state of cells, tissues, and organisms under specific physiological conditions (5). Unlike genomic or transcriptomic studies, metabolomic studies capture real biochemical phenomena and provide a unique view of the physiological processes that lead to the reproductive transitions (6). Ultra-high-performance liquid chromatography combined with tandem mass spectrometry (UHPLC-MS/MS) has become a gold-standard platform for untargeted serum metabolomics in animal research, providing high sensitivity, wide metabolite coverage, and reliable identification through MS2 fragmentation matching (7). Recent studies using serum metabolomics in small ruminants have shown that the serum metabolome changes substantially across reproductive states, and that lipids, amino acids, and energy-related metabolites serve as central biomarkers of reproductive transitions (8, 9).

Among the metabolic pathways regulating female reproductive function, choline metabolism and carnitine metabolism have attracted particular attention. Choline is an essential nutrient and an important precursor of phosphatidylcholine, which is a major component of the eukaryotic cell membrane. Phosphatidylcholines accumulate gradually during the growth of follicles, and their depletion severely impairs the development of the follicles and the formation of the antrum (10). In addition, choline is a substrate for the synthesis of betaine, a key methyl donor that plays a role in one-carbon metabolism, and glycerophosphocholine, which is an organic osmolyte in reproductive tissues (10, 40). L-carnitine and its acetylated derivative play an essential role in female reproductive physiology by facilitating the transport of long-chain fatty acids into mitochondria in the process of β-oxidation, thereby supporting oocyte energy metabolism, maintaining cell membrane stability through phospholipid acetylation, and reducing oxidative stress in the reproductive system (11). Choline and carnitine metabolites have been identified as important indicators of the reproductive metabolic status in ruminants, but their dynamic changes across the reproductive stages of hormone-induced estrus in ewes have not yet been systematically characterized.

The aim of this study was to characterize the serum metabolome of Kazak ewes at three time points within the CIDR-PGF_2α_-induced estrus using UHPLC-MS/MS-based untargeted metabolomics. The three time points are the luteal phase (LP), the luteolysis phase (LDP), and the estrus phase (OES). In particular, we aimed to identify significant differences in metabolites and enriched metabolic pathways between the pair comparisons of LP, LDP, and OES, with a particular focus on whether choline and carnitine related metabolites show significant stage- dependent changes. Our results offer a comprehensive characterization of the metabolic dynamics of the serum associated with hormone induced estrus in ewes and provide a metabolic basis for future research incorporating naturally induced estrus metabolomics and nutritional intervention strategies targeting key reproductive metabolic pathways.

## 2 Materials and methods

### 2.1 Ethics approval

All experiments involving animals received approval from the Biology Ethics Committee of Shihezi University (Shihezi, China) under approval number A2024-442. Blood samples were collected by qualified veterinarians. The research was conducted in full accordance with Directive 2010/63/EU on the protection of animals used for scientific purposes.

### 2.2 Animals and sample collection

Eighteen Kazak ewes aged 3–4 years, with an average body weight of 41.1 ± 2.3 kg, were housed individually in well-ventilated pens at an experimental farm in Shihezi, Xinjiang, China, with *ad libitum* access to clean water. The basic diet consisted of commercial concentrates (Tian Kang 972, China) as the primary nutrient source, with alfalfa hay and corn stalks as roughage at a concentrate-to-roughage ratio of approximately 30:70. Each animal received 0.20–0.22 kg of concentrates and 0.48–0.52 kg of roughage daily (alfalfa hay 70%, corn stalks 30%), and was fed twice daily at 09:00 and 19:00 (Xinjiang local time) with concentrates offered before roughage. Prior to the experiment, standard pest control measures were applied to all animals in accordance with farm management protocols.

All ewes were synchronized using the CIDR-PGF_2α_ hormone protocol to induce estrus (3). The reproductive stage at each sampling time point was defined by the CIDR-PGF_2α_ protocol schedule; the expected hormonal dynamics across these stages, including elevated serum progesterone (P4) during the luteal phase, declining P4 at luteolysis, and rising estradiol (E2) at estrus, have been previously characterized in Kazak ewes of the same breed using ELISA-based hormonal assays under induced reproductive conditions in our laboratory (4). Serum samples were collected at three defined time points corresponding to distinct reproductive stages: the luteal phase (LP), the luteolysis phase (LDP), and the estrus phase (OES), with six ewes per group (*n* = 6). Blood samples (5 ml) were collected from the jugular vein of each ewe by trained veterinarians and transferred directly into vacuum collection tubes. Samples were centrifuged to obtain serum and immediately stored at −80 °C until metabolomics analysis.

### 2.3 Serum sample preparation

Serum metabolites were extracted using an automated protein precipitation method. Briefly, a 96-well protein precipitation plate and collection plate combination was placed into a Starlid™ automated workstation. Prior to extraction, a mixture of deuterated internal standards was added to the extraction solution to monitor extraction efficiency, instrument signal stability, and analytical reproducibility throughout the entire run. Then, 100 μl of serum was added to the 96-well protein precipitation plate, followed by 400 μl of pre-cooled (−40 °C) extraction solution (methanol:acetonitrile, 1:1, v/v) containing the deuterated internal standards (12). The mixture was shaken at 750 rpm for 5 min, allowed to stand for 5 min, and then filtered to collect the filtrate. To assess instrument stability and analytical reproducibility, quality control (QC) samples were prepared by pooling equal volumes of filtrate from all 18 samples. A total of three QC samples were prepared and injected at regular intervals throughout the analytical run to monitor system stability and signal consistency.

### 2.4 UHPLC-MS/MS analysis

Metabolomics profiling was performed using a UHPLC system (Vanquish, Thermo Fisher Scientific, Waltham, MA, USA) coupled to an Orbitrap Exploris 120 high-resolution mass spectrometer (Thermo Fisher Scientific) (3). For chromatographic separation of polar and semi-polar metabolites, a Waters ACQUITY UPLC BEH Amide column (2.1 mm × 50 mm, 1.7 μm) was used. This column configuration was optimized for the detection of polar metabolites including amino acids, organic acids, nucleotides, and polar lipids, whereas coverage of non-polar lipid species, such as neutral lipids and triglycerides, was consequently limited, which represents a recognized constraint of the current analytical platform. The mobile phase was composed of two solvents: mobile phase A contained 25 mmol/L ammonium acetate and 25 mmol/L ammonium hydroxide in water (pH 9.75), while mobile phase B was acetonitrile. The auto-sampler temperature was maintained at 4 °C, and each sample injection volume was 2 μl. Each biological sample was injected once into the instrument, consistent with standard practice for untargeted metabolomics; analytical reproducibility and instrument stability were monitored throughout the run using pooled QC samples injected at regular intervals, as described in Section 2.3. Data acquisition was performed in both positive and negative electrospray ionization modes using Xcalibur software (version 4.4, Thermo Fisher Scientific) operating in information-dependent acquisition (IDA) mode. The ESI source parameters were configured as follows: sheath gas flow rate of 50 Arb; auxiliary gas flow rate of 15 Arb; capillary temperature of 320 °C; full MS resolution of 60,000; MS/MS resolution of 15,000; stepped normalized collision energy (SNCE) set to 20, 30, and 40 eV; spray voltages of +3.8 kV for positive mode and −3.4 kV for negative mode (3).

### 2.5 Data preprocessing and quality control

Raw data files were converted to mzXML format using ProteoWizard software (version 3.0.24054). Feature detection, extraction, alignment, and integration were performed using an in-house R program based on XCMS. Metabolite identification was conducted by matching MS^1^ accurate mass and MS^2^ fragmentation patterns against an in-house database (13). Putative identifications were further cross-validated against two public databases, the Human Metabolome Database (HMDB), from which 1,016 metabolites received HMDB identifiers, and the KEGG compound database for Ovis aries, from which 515 metabolites received KEGG identifiers, providing additional annotation confidence beyond the in-house spectral library alone. Initially, 31,918 features were extracted, following preprocessing steps: (1) outlier filtering based on relative standard deviation (RSD), whereby features exhibiting an RSD greater than 30% across the three QC samples were removed to eliminate analytically unstable signals; (2) missing value filtering, retaining only features with no more than 50% missing values within any single group; (3) missing value imputation, using half of the minimum detected value for each feature to replace remaining missing entries; and (4) dual normalization, combining total ion current (TIC)-based normalization of each sample with internal standard correction using the six deuterated internal standards to simultaneously account for sample-to-sample loading variation and instrument signal drift. Following this preprocessing pipeline, 31,912 features were retained. Among these, 1,861 metabolites were annotated at the MS^2^ level. Complete lists of detected metabolites in positive and negative electrospray ionization modes are provided in Supplementary Tables S1, S2, respectively. The Metabolomics Standards Initiative (MSI) classification system was used to assign confidence levels to each identified metabolite (14). Level 1 metabolites were confirmed by MS^1^ accurate mass, MS^2^ fragmentation spectrum, and chromatographic retention time matching against reference standards, yielding 537 confirmed metabolites. Level 2 metabolites were putatively annotated by MS^1^ and MS^2^ spectral matching, yielding 1,233 putatively annotated metabolites. Level 3 metabolites were putatively characterized by MS1 accurate mass only, yielding 91 metabolites. Level 4 metabolites remained unknown, accounting for 30,051 of the total 31,912 detected features. Only metabolites classified as Level 1 and Level 2 were included in the Results and Discussion sections, as these represent annotation of sufficient confidence for biological interpretation. Level 3 and Level 4 metabolites were excluded from downstream analysis. The proportion of Level 4 unknowns reflects an inherent limitation of current MS^2^ spectral library coverage. Three QC samples were analyzed to monitor the analytical quality throughout the experiment, each prepared by pooling equal volumes of all 18 experimental sample filtrates, and injected at regular intervals across the complete analytical run. All three QC samples clustered tightly within ±2 standard deviations in the PCA score plot, confirming the reproducibility and stability of the analytical platform. QC sample correlation coefficients exceeded 0.85 across all comparisons, providing additional confirmation of method stability throughout the run. The RSD of all six deuterated internal standards across the three QC samples was below 1% for every standard (range: 0.10%−0.64%), confirming high instrument stability throughout the run. Furthermore, blank samples interspersed during the run showed no detectable internal standard peaks, confirming that cross-sample carryover was effectively controlled.

### 2.6 Multivariate statistical analysis

Multivariate statistical analyses were performed using SIMCA software (version 18.0.1, Sartorius Stedim Data Analytics AB, Umea, Sweden) (3). Principal component analysis (PCA) was applied using log_10_ transformation and centering (CTR) scaling to evaluate global metabolic variation and analytical reproducibility. Orthogonal partial least-squares discriminant analysis (OPLS-DA) was then performed using log_10_ transformation and unit variance (UV) scaling to identify the metabolites that contributed most to differences between groups. Model robustness was evaluated using 7-fold cross-validation, and model validity was confirmed by 200-permutation testing. All Q^2^ intercept values were negative, confirming no overfitting (3).

### 2.7 Differential metabolite identification

Significantly differential metabolites between each pair of comparison groups (LP vs. LDP, LP vs. OES, and LDP vs. OES) were identified using two combined criteria. First, the variable importance in projection (VIP) from the OPLS-DA model had to be greater than 1.0. Secondly, the *p*-value from Student's *t*-test had to be less than 0.05 (3, 15). Each of the three comparisons was conducted as an independent two-group analysis, as this pairwise design was most appropriate for characterizing sequential metabolic transitions between defined reproductive stages. Fold change for each metabolite in each comparison was calculated as the mean abundance in the second group divided by the mean abundance in the first group in each pairwise comparison. Finally, differential metabolites were visually displayed using volcano plots. Volcano plots were generated using Python (version 3.12.3) with the matplotlib (version 3.10.8) and adjustText (version 1.4.0) libraries.

### 2.8 Hierarchical cluster analysis and heatmap visualization

To visualize how metabolic patterns changed across the different reproductive stages, hierarchical cluster analysis (HCA) was performed on the top 30 differential metabolites by VIP score from each of the three pairwise comparisons (LP vs. LDP, LP vs. OES, and LDP vs. OES). This selection yielded 66 unique metabolites in total, and only those with identification confidence levels 1 and 2 were included. Before clustering, the abundance values for each metabolite were normalized across rows to Z-scores. Hierarchical clustering was then applied to the metabolite rows using Ward.D2 linkage. Finally, heatmaps were created in R (version 4.5.2) using the pheatmap package (version 1.0.13), and color scales were defined using the RColorBrewer package (version 1.1.3).

### 2.9 KEGG pathway enrichment analysis

To identify which metabolic pathways were involved in the stage-dependent alterations observed in serum metabolites, KEGG pathway enrichment analysis was performed for each pairwise comparison using the kyoto encyclopedia of genes and genomes (KEGG) database for *Ovis aries* (sheep) (16, 17). For each pathway, the rich factor was calculated as the number of differential metabolites that mapped to that pathway divided by the total number of detected metabolites that were annotated in that pathway. Fisher's exact test was used to determine whether each pathway was enriched, and pathways with a *p* < 0.05 were considered significant. Enriched pathways were visualized as bubble plots, where the size of the bubble reflects the number of differential metabolites mapped to each pathway, and the intensity of the bubble's color indicates the statistical significance (3).

## 3 Results

### 3.1 Overview of serum metabolic profiles

**Figure 1:**
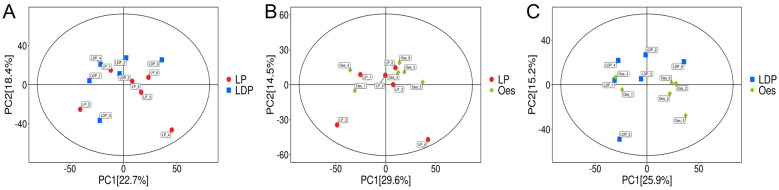
The score plots of Principal component analysis (PCA) of serum metabolic profiles across three reproductive stages. **(A)** LP vs. LDP: PC1 = 22.7%, PC2 = 18.4%; **(B)** LP vs. OES: PC1 = 29.6%, PC2 = 14.5%; **(C)** LDP vs. OES: PC1 = 25.9%, PC2 = 15.2%. Each point represents one animal (*n* = 6 per group). QC samples are shown as gray dots and cluster tightly together, indicating excellent analytical reproducibility and stability.

To evaluate global metabolic differences among the three reproductive stages, principal component analysis (PCA) was performed on serum samples collected from ewes at the luteal phase (LP), luteolysis phase (LDP), and estrus phase (OES) (Figures 1A–C). In the LP vs. LDP comparison, PC1 and PC2 explained 22.7 and 18.4% of the total variance, respectively, with partial separation observed between the two groups, reflecting the close physiological relationship between these adjacent stages. In the LP vs. OES comparison, PC1 and PC2 accounted for 29.6 and 14.5% of the total variance, respectively, with the two groups separating primarily along PC1. In the LDP vs. OES comparison, the most pronounced separation was observed, with PC1 and PC2 explaining 25.9 and 15.2% of the total variance, respectively, indicating that LDP and OES represent the most metabolically distinct reproductive stages in the hormone-induced estrus process. In all comparisons, quality control (QC) samples clustered tightly together, confirming the stability and reproducibility of the analytical platform throughout the experiment.

### 3.2 Multivariate statistical analysis

**Figure 2:**
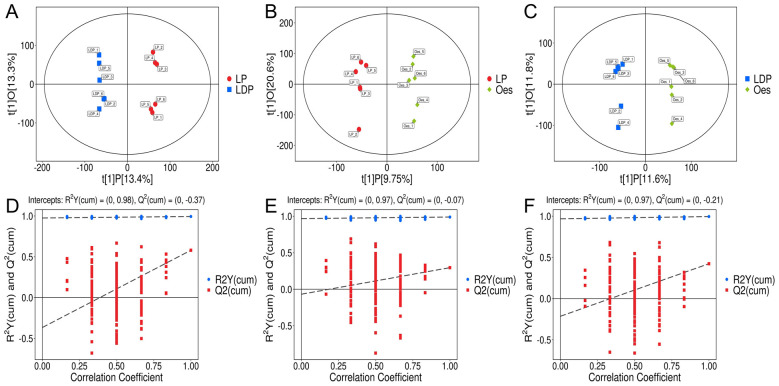
OPLS-DA score plots and the results of permutation tests for three pairwise comparisons. **(A–C)** The OPLS-DA score plots for LP vs. LDP, LP vs. OES, and LDP vs. OES, respectively. **(D–F)** The corresponding 200-permutation validation plots. Each model shows the values (blue dots: R^2^Y, red squares: Q^2^). Negative Q^2^ intercepts in all permutation tests confirm model robustness and the absence of overfitting.

To identify differential metabolites between reproductive stages, orthogonal partial least squares discriminant analysis (OPLS-DA) was performed for all three pairwise comparisons. In the comparison of LP and LDP, the OPLS-DA model showed complete separation between the two groups along the predicted component (*t* (1)*P* = 13.4%), with *R*^2^*Y* = 0.994 and *Q*^2^ = 0.580, confirming strong model performance. The comparison of LP and OES displayed clear group separation along both the predicted components (*t* (1)*P* = 9.75%) and orthogonal (*t* (1)*O* = 20.6%), with *R*^2^*Y* = 0.989 and *Q*^2^ = 0.296. The LDP and OES comparison achieved the most complete separation between groups with the highest model reliability among the three comparisons (*R*^2^*Y* = 0.995, *Q*^2^ = 0.423). To validate all models and exclude overfitting, 200-fold permutation tests with 7-fold cross-validation were performed. In all permutation tests, *Q*^2^ intercept values were negative (LP vs. LDP ≈ −0.37, LP vs. OES ≈ −0.07, LDP vs. OES ≈ −0.21), confirming that the metabolic separation observed between reproductive stages is statistically robust and is not random (Figures 2A–F).

### 3.3 Significantly different metabolites between the three comparisons

**Figure 3:**
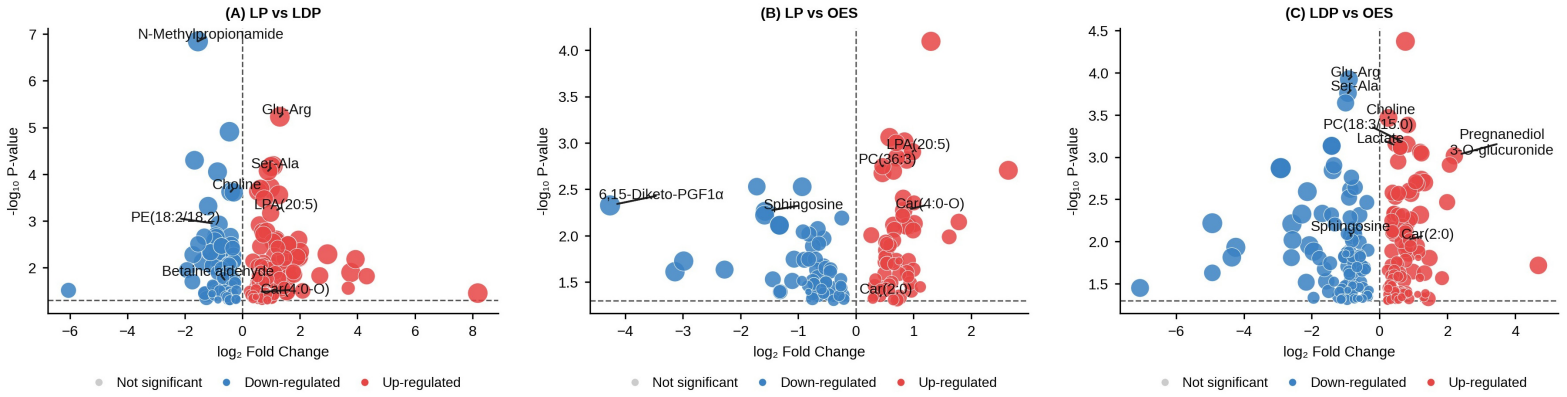
Volcano plots of differentially abundant serum metabolites in hormone-induced estrus transitions in ewes. Comparisons are shown for each pair **(A)** LP vs. LDP, **(B)** LP vs. OES, and **(C)** LDP vs. OES. The red dots represent significantly higher metabolites, the blue dots represent lower metabolites, and the gray dots indicate metabolites with no significant differences. The diameter of the each circle is proportional to the Variable Importance in Projection (VIP) score. The horizontal dashed line indicates the statistical significance threshold of *P* < 0.05 (–log_10_ *P*-value > 1.30), and the vertical dashed line indicates log_2_ (Fold Change) = 0, separating upregulated and downregulated metabolites.

Differentially abundant metabolites were identified using combined criteria: VIP > 1.0 from the OPLS-DA model and *p* < 0.05 from Student's *t*-test. The significantly different metabolites were visualized using volcano plots. The figure clearly shows that numerous metabolites between each pair of groups were significantly different. A total of 213, 119, and 203 differentially abundant metabolites were identified in the LP vs. LDP, LP vs. OES, and LDP vs. OES comparisons, respectively (Figure 3; Table 1).

**Table 1:** Summary of differentially abundant metabolites identified across three reproductive stages in CIDR-PGF_2_α-treated ewes.

Comparison	Total detected	Differentially abundant metabolites	Upregulated	Downregulated
LP vs. LDP	1,861	213	121	92
LP vs. OES	1,861	119	61	58
LDP vs. OES	1,861	203	104	99

Differentially abundant metabolites were identified using combined criteria: VIP > 1.0 from OPLS-DA models and *p* < 0.05 from Student's *t*-test. Upregulated and downregulated are relative to the second group in each comparison.

### 3.4 Identification of differentially abundant metabolites in LP vs. LDP

In total, 213 significantly different metabolites were identified between LP vs. LDP. Compared with the LP stage, 121 metabolites had higher concentrations in LDP, while 92 were reduced. Among the increased metabolites, amino acid-related dipeptides were prominently represented, including Glu-Arg (FC = 2.468) and Ser-Ala (FC = 1.858), indicating enhanced amino acid turnover during the onset of luteolysis. LPA (20:5) (FC = 1.976) and PC (36:3) (FC = 1.471) also increased, reflecting early glycerophospholipid remodeling. The short-chain acylcarnitine 3-hydroxybutyrylcarnitine [Car (4:0-O)] was elevated in LDP (FC = 1.284), suggesting early activation of fatty acid β-oxidation. Among the decreased metabolites, choline (FC = 0.753), betaine aldehyde (FC = 0.728), 1,2-dilinoleoyl-sn-glycero-3-phosphoethanolamine (FC = 0.570), and N-Methylpropionamide (FC = 0.343) showed marked reductions (Supplementary Table S3).

### 3.5 Identification of differentially abundant metabolites in LP vs. OES

In the LP vs. OES comparison, 119 significantly different metabolites were identified, with 61 higher and 58 lower in OES relative to LP. Metabolites elevated in OES included LPA (20:5) (FC = 1.972), PC (36:3) (FC = 1.378), 3-hydroxybutyrylcarnitine (Car(4:0-O)] (FC = 1.775), and acetyl carnitine [Car (2:0)] (FC = 1.341). In contrast, 6,15-diketo-13,14-dihydroprostaglandin F1α (FC = 0.052) and sphingosine (FC = 0.336) decreased markedly in OES (Supplementary Table S4).

### 3.6 Identification of differentially abundant metabolites in LDP vs. OES

In the LDP vs. OES comparison, 203 significantly different metabolites were identified, with 104 higher and 99 lower in OES relative to LDP. Among the increased metabolites in OES, pregnanediol 3-O-glucuronide showed the most pronounced change (FC = 4.600), followed by PC (18:3(6Z, 9Z, 12Z)/15:0) (FC = 1.757), choline (FC = 1.198), glycerophosphocholine (FC = 1.275), lactate (FC = 1.522) and acetyl carnitine [Car (2:0)] (FC = 1.560). Conversely, Glu-Arg (FC = 0.533), Ser-Ala (FC = 0.524), and sphingosine (FC = 0.556) were reduced in OES (Supplementary Table S5).

The key differentially abundant metabolites identified in the three comparisons, including their VIP scores, *p*-values, fold changes, and regulatory trends, are summarized in Table 2.

**Table 2:** Key differentially abundant metabolites identified across three reproductive stage comparisons in CIDR-PGF_2α_-treated ewes.

ID	Metabolite	Type	VIP	*p*-value	Fold change	Trend	Comparison	Level
803	N-Methylpropionamide	POS	2.548	1.43 × 10^−7^	0.343	**↓**	LP vs. LDP	Level2
429	Glu-Arg	POS	2.507	5.89 × 10^−6^	2.468	**↑**	LP vs. LDP	Level1
257	Ser-Ala	NEG	2.396	8.47 × 10^−5^	1.858	**↑**	LP vs. LDP	Level1
238	Choline	POS	2.391	2.37 × 10^−4^	0.753	**↓**	LP vs. LDP	Level1
942	1,2-Dilinoleoyl-sn-glycero-3-phosphoethanolamine	POS	2.22	1.17 × 10^−3^	0.57	**↓**	LP vs. LDP	Level2
1051	LPA (20:5)	POS	2.21	6.83 × 10^−4^	1.976	**↑**	LP vs. LDP	Level2
325	Betaine aldehyde	POS	1.786	2.38 × 10^−2^	0.728	**↓**	LP vs. LDP	Level1
351	3-Hydroxybutyrylcarnitine [Car (4:0-O)]	POS	1.67	3.37 × 10^−2^	1.284	**↑**	LP vs. LDP	Level1
686	6,15-Diketo-13,14-dihydroprostaglandin F1α	NEG	3.045	4.71 × 10^−3^	0.052	**↓**	LP vs. OES	Level2
367	Sphingosine	POS	2.831	5.45 × 10^−3^	0.336	**↓**	LP vs. OES	Level1
1051	LPA (20:5)	POS	2.576	1.25 × 10^−3^	1.972	**↑**	LP vs. OES	Level2
351	3-Hydroxybutyrylcarnitine [Car (4:0-O)]	POS	2.58	5.35 × 10^−3^	1.775	**↑**	LP vs. OES	Level1
402	Acetyl carnitine [Car (2:0)]	POS	1.844	4.47 × 10^−2^	1.341	**↑**	LP vs. OES	Level1
712	PC (36:3)	POS	2.429	1.78 × 10^−3^	1.378	**↑**	LP vs. OES	Level2
429	Glu-Arg	POS	2.564	1.20 × 10^−4^	0.533	**↓**	LDP vs. OES	Level1
238	Choline	POS	2.555	3.44 × 10^−4^	1.198	**↑**	LDP vs. OES	Level1
257	Ser-Ala	NEG	2.531	1.74 × 10^−4^	0.524	**↓**	LDP vs. OES	Level1
205	Pregnanediol 3-O-glucuronide	NEG	2.491	9.64 × 10^−4^	4.6	**↑**	LDP vs. OES	Level1
1429	PC (18:3(6Z,9Z,12Z)/15:0)	POS	2.417	7.04 × 10^−4^	1.757	**↑**	LDP vs. OES	Level2
368	Lactate	NEG	2.235	7.69 × 10^−4^	1.522	**↑**	LDP vs. OES	Level1
402	Acetyl carnitine [Car (2:0)]	POS	2.129	9.92 × 10^−3^	1.56	**↑**	LDP vs. OES	Level1
367	Sphingosine	POS	2.304	8.59 × 10^−3^	0.556	**↓**	LDP vs. OES	Level1

The “↑” indicates upregulation and “↓” indicates downregulation of the second group in each comparison. VIP values were obtained from OPLS-DA models (VIP > 1.0); *p*-values were calculated from Student's *t*-test, *p* < 0.05. Fold change = mean of second group / mean of first group. POS, positive ESI mode; NEG, negative ESI mode; Level 1, confirmed identification; Level 2, putative identification. Rows highlighted in yellow indicate acylcarnitine metabolites.

### 3.7 Shared differentially abundant metabolites across the three comparisons

Sphingosine, choline, 3-hydroxybutyrylcarnitine [Car (4:0-O)], acetyl carnitine [Car (2:0)], and PC (36:3) were significant metabolites shared across the pairwise comparisons. Especially, sphingosine was consistently downregulated in all three comparisons. Choline and 3-hydroxybutyrylcarnitine were shared significant metabolites in LP vs. LDP and their respective OES comparisons. The acetyl carnitine [Car (2:0)] and PC (36:3) were shared in two comparisons each (Table 3).

**Table 3:** Shared differentially abundant metabolites across pairwise comparisons of reproductive stages in CIDR-PGF_2_α-treated ewes.

Metabolite	Molecular formula	Comparison	Fold change^a^	*p*-value^b^	VIP^c^	Up/down^d^
Sphingosine	*C_18_H_37_NO_2_*	LP vs. LDP	0.604	0.0326	1.712	Down
		LP vs. OES	0.336	0.0055	2.831	Down
		LDP vs. OES	0.556	0.0086	2.304	Down
Choline	*C_5_H_14_NO*	LP vs. LDP	0.753	0.0002	2.391	Down
		LDP vs. OES	1.198	0.0003	2.555	Up
3-Hydroxybutyrylcarnitine [Car (4:0-O)]	*C_11_H_21_NO_5_*	LP vs. LDP	1.284	0.0337	1.670	Up
		LP vs. OES	1.775	0.0054	2.580	Up
Acetyl carnitine [Car (2:0)]	*C_9_H_17_NO_4_*	LP vs. OES	1.341	0.0447	1.844	Up
		LDP vs. OES	1.560	0.0099	2.129	Up
PC (36:3)	*C_44_H_82_NO_8_P*	LP vs. LDP	1.471	0.0432	1.807	Up
		LP vs. OES	1.378	0.0018	2.429	Up

^a^Fold change = mean of second group / mean of first group.

^b^*p*-values were derived from Student's *t*-test for each independent pairwise comparison.

^c^Variable Importance in Projection from OPLS-DA model (VIP > 1.0).

^d^Compared with the first group of each comparison, the second group presents upregulation or downregulation of this metabolite.

**Figure 4:**
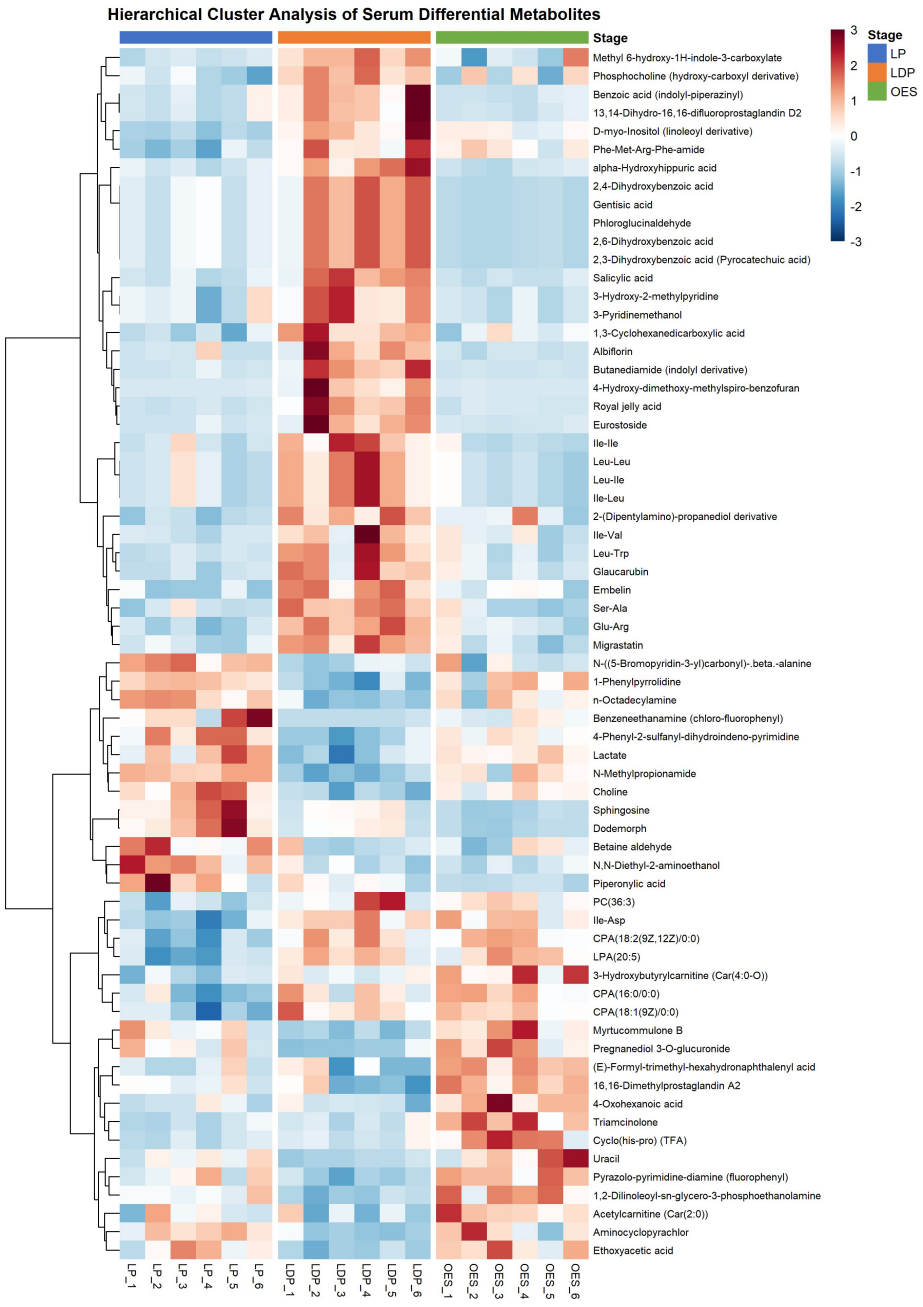
Hierarchical cluster analysis (HCA) heatmap of significantly different serum metabolites across three reproductive stages in CIDR-PGF_2α_-treated ewes. Rows represent 66 differentially abundant serum metabolites selected from the top 30 metabolites by VIP score from each pairwise comparison (LP vs. LDP, LP vs. OES, and LDP vs. OES; Level 1 and Level 2 identifications only), and columns represent individual animals (*n* = 6 per group). Abundance values are presented as row-normalized Z-scores. Red indicates relatively higher abundance, blue indicates relatively lower abundance, and white indicates abundance close to the group mean. Metabolite rows were clustered using Ward.D2 hierarchical clustering. Vertical lines separate the three reproductive stages: luteal phase (LP, blue), luteolysis phase (LDP, orange), and estrus phase (OES, green).

Hierarchical cluster analysis of the top differentially abundant metabolites across the three reproductive stages revealed clear stage-dependent metabolic patterns (Figure 4). Samples clustered primarily according to their physiological stage, with LP and OES forming distinct clusters and LDP occupying an intermediate position. Choline and glycerophosphocholine-related metabolites exhibited relatively high abundance in LP, declined markedly in LDP, and recovered in OES. Acylcarnitine species, particularly acetyl carnitine and 3-hydroxybutyrylcarnitine, showed progressive increases from LP toward OES. Amino acid-related dipeptides including Glu-Arg and Ser-Ala peaked during LDP and declined in OES, while steroid-related metabolites and glycolytic intermediates were most elevated at the OES stage.

### 3.8 KEGG pathway enrichment analysis

**Figure 5:**
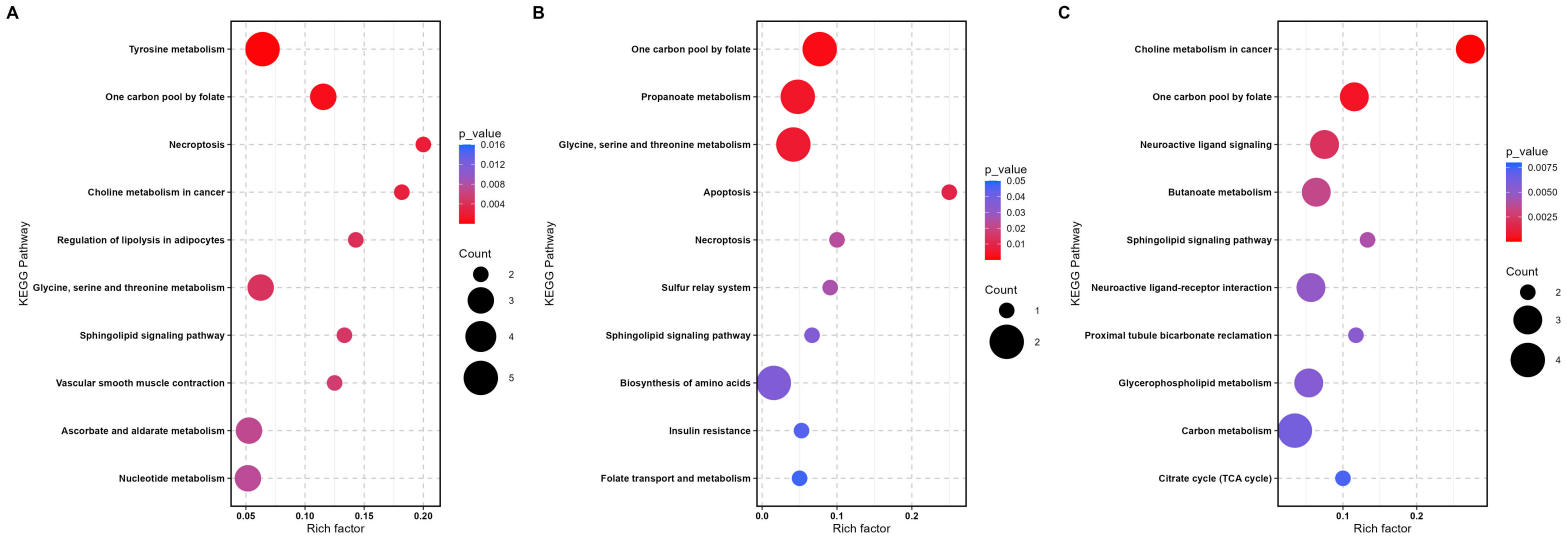
KEGG pathway enrichment analysis of significantly different serum metabolites across three reproductive stages in CIDR-PGF_2α_-treated ewes. Bubble plots illustrating enriched KEGG pathways for each pairwise comparison: **(A)** LP vs. LDP, **(B)** LP vs. OES, and **(C)** LDP vs. OES. Each bubble represents one enriched pathway. The x-axis indicates the rich factor (ratio of differentially abundant metabolites to total detected metabolites within the pathway). Bubble size reflects the number of differentially abundant metabolites mapped to each pathway, and bubble color represents the *p*-value of enrichment, with red indicating higher statistical significance and blue indicating lower statistical significance. Only pathways with *p* < 0.05 are shown. LP, luteal phase; LDP, luteolysis phase; OES, estrus phase. Full pathway enrichment statistics for all significant pathways are provided in Supplementary Table S6.

To identify the metabolic pathways underlying the stage-dependent serum metabolic changes of the three reproductive phases, KEGG pathway enrichment analysis was conducted for each pair comparison. Enriched pathways are displayed as bubble plots (Figure 5), and the size of each bubble reflects the number of differentially abundant metabolites mapped to each pathway, the x-axis represents the rich factor, and the intensity of the bubble's color indicates the statistical significance.

In the comparison of LP and LDP, 79 KEGG pathways were identified, 15 of which had statistical significance (*p* < 0.05) (Figure 5A). The most prominent enriched pathways included a one-carbon pool by folate (rich factor = 0.115, *p* < 0.001), choline metabolism (rich factor = 0.182, *p* = 0.003), glycine, serine, and threonine metabolism (rich factor = 0.062, *p* = 0.004), and sphingolipid signaling pathway (rich factor = 0.133, *p* = 0.005). One-carbon pool by folate and glycine, serine, and threonine metabolism were enriched through choline (C00114), betaine aldehyde (C00576) and creatine (C00300), all significantly downregulated in LDP relative to LP. Choline metabolism was enriched through choline and 1,2-dilinoleoyl-sn-glycero-3-phosphoethanolamine (C04230). The sphingolipid signaling pathway was enriched through sphingosine (C00319) and adenosine (C00212), both of which were downregulated in LDP.

In a comparison of LP and OES, 28 KEGG pathways were identified, 11 of which had statistical significance (*p* < 0.05) (Figure 5B). The most significant pathways of enrichment were one-carbon pool by folate (rich factor = 0.077, *p* = 0.002), propanoate metabolism (rich factor = 0.048, *p* = 0.004), glycine, serine, and threonine metabolism (rich factor = 0.042, *p* = 0.006), apoptosis (rich factor = 0.250, *p* = 0.010), and sphingolipid signaling pathway (rich factor = 0.067, *p* = 0.036). Propanoate metabolism was enriched through 3-hydroxyisobutyrate (C05984) and lactaldehyde (C00546), both of which gradually increased toward OES. Apoptosis was enriched exclusively by sphingosine (C00319), which was significantly decreased in Oes compared to LP (fold change = 0.336, *p* = 0.006). One-carbon pool by folate and glycine, serine, and threonine metabolism were enriched by betaine aldehyde (C00576) and S-adenosyl-L-methionine (C00019), both significantly reduced from LP to OES.

In the LDP and OES comparison, 101 KEGG pathways were identified, the largest number in the three comparisons, of which 15 were statistically significant (*p* < 0.05) (Figure 5C). The most significantly enriched pathways were choline metabolism (rich factor = 0.273, *p* < 0.001), one-carbon pool by folate (rich factor = 0.115, *p* < 0.001), butanoate metabolism (rich factor = 0.064, *p* = 0.003), glycerophospholipid metabolism (rich factor = 0.054, *p* = 0.006), and carbon metabolism (rich factor = 0.035, *p* = 0.006). Choline metabolism displayed the highest rich factor across all three comparisons, enriched through choline (C00114), glycerophosphocholine (C00670), and 1,2-dilinoleoyl-sn-glycero-3-phosphoethanolamine (C04230), all of which are significantly upregulated in OES compared to LDP. Glycerophospholipid metabolism was enriched by the same choline-containing metabolites. Butanoate metabolism was enriched through 3-hydroxybutyrylcarnitine [Car (4:0-O)] and acetyl carnitine [Car (2:0)], both of which were significantly increased at OES. Carbon metabolism was enriched by lactate (C00184) and uracil (C00232).

In all three comparisons, one-carbon pool was consistently enriched by folate and the sphingolipid signaling pathway, indicating sustained alterations in choline-mediated one-carbon metabolism and sphingolipid signaling during the LP–LDP–OES transition. Choline metabolism and glycerophospholipid metabolism showed the strongest enrichment at the LDP to OES stage, while the progressive enrichment of propanoate metabolism and butanoate metabolism from LP to OES reflects a phase-dependent increase in fatty acid β-oxidation facilitated by carnitine as ewes approach estrus (Figure 5; Supplementary Table S6).

## 4 Discussion

The metabolomics findings of this study are correlational observations that can provide direction for future validation studies, rather than providing direct causal evidence for the molecular mechanisms described. Reproductive transitions in livestock, such as puberty, estrus, pregnancy, or lactation, are driven by a complex cascade of hormones. However, the effects of these hormonal changes on the body's metabolism go far beyond simply raising or lowering the levels of well-known hormones such as progesterone and estrogen. Analyzing the full set of small molecules in blood, termed Serum metabolomics, is now increasingly recognized as a powerful tool for tracking real-time biochemical changes occurring in livestock during reproductive events(18). In the present study, UHPLC-MS/MS-based untargeted metabolomics identified 213, 119, and 203 differentially abundant metabolites in the LP vs. LDP, LP vs. OES, and LDP vs. OES comparisons. KEGG pathway enrichment analysis revealed coordinated alterations across one-carbon metabolism, fatty acid β-oxidation, sphingolipid signaling, and glycerophospholipid biosynthesis. The OPLS-DA models showed good separation between groups, with R^2^Y values of 0.994, 0.989, and 0.995. Additionally, all permutation Q^2^ intercepts were negative, confirming real biological differences between reproductive stages without model overfitting. We compared all three pairwise comparisons and found 15 out of 101 significantly enriched KEGG pathways during the transition from LDP to OES. Furthermore, the multivariate group separation showed no overlap as progesterone breaks down, the corpus luteum collapses, and follicular activation accelerates toward ovulation. These results suggest that serum metabolomics can capture key internal changes during the hormone induced-estrus transitions in Kazak ewes and may provide a useful platform for the identification of reproductive stage biomarkers. However, it is important to interpret these findings with caution. The Q^2^ values from the OPLS-DA models ranged from 0.296 to 0.580, indicating moderate predictive ability rather than excellent discrimination for unknown data. This, combined with the relatively small sample size (*n* = 6 per group), suggests that the present results should be considered preliminary. Validation in larger cohorts is warranted to confirm the stage-dependent metabolic patterns observed in this study.

### 4.1 Choline metabolism and one-carbon pool by folate are central to the LP–LDP–OES transition

In the present study, we found that choline metabolism was the most significantly enriched pathway in the LP and LDP comparison (RF = 0.182, *p* = 0.003), and even more enriched when comparing the LDP and OES (RF = 0.273, *p* < 0.001). It achieved the highest rich factor of any pathway across all three comparisons. Another related process, called one-carbon pool by folate, was the only KEGG pathway enriched in all three pairwise comparisons, consistently driven by choline (C00114), betaine aldehyde (C00576), and S-adenosyl-L-methionine (SAM, C00019). These results suggest that choline-mediated one-carbon metabolism is not merely altered but changes in a steady, coordinated manner throughout the hormone-induced estrus process.

Among the key differentially abundant metabolites, choline itself changed in a consistent manner across the three stages. In the middle stage LDP, choline levels were significantly reduced compared to the early stage LP (FC = 0.753, *p* = 0.0002, VIP = 2.391) and then partially recovered in OES relative to LDP (FC = 1.198, *p* = 0.0003, VIP = 2.555). Two related substances, betaine aldehyde (the immediate oxidation product of choline), were also reduced in LDP compared to LP, while another substance, glycerophosphocholine, was significantly higher in the late-stage OES relative to LDP (FC = 1.275). The lower levels of circulating choline and betaine aldehyde during the middle stage LDP likely reflect increased utilization by peripheral tissues. At that time, the corpus luteum is breaking down, a process that requires choline for cell membrane breakdown, triggering cell death signals and rearrangement of genetic material. As a result, less choline is available in the bloodstream. Choline is an essential nutrient that supports the synthesis of phospholipid membranes, donates methyl group via betaine, and produces acetylcholine, a neurotransmitter (19). Thus, the lower choline levels in the LDP are not merely a random fluctuation but likely reflect increased tissue utilization to support major physiological remodeling. These results are consistent with findings in sheep serum metabolomics, where choline-related metabolites also showed predictable changes across the reproductive cycle, reflecting the body's increased demand for metabolic precursors as the animal transitions between reproductive phases (20).

The fact that the one-carbon pool by folate remained active across all three comparisons reflects that this pathway plays a central role in supporting methylation reactions during reproductive transitions. In this pathway, choline is first converted into betaine through oxidation; betaine then transfers a methyl group with the help of the enzyme betaine-homocysteine methyltransferase. The transfer of the methyl group converts homocysteine into methionine, subsequently regenerating SAM, the universal methyl group donor for epigenetic modifications, including DNA and histone methylation (21). SAM was identified as one of the contributing metabolites to the enrichment of this pathway when comparing the early-stage LP to the late-stage OES, where SAM level was significantly reduced toward the late-stage OES along with betaine aldehyde. This temporary suppression of methylation capacity during middle stage LDP and the start of late-stage OES likely reflects a period of intense epigenetic reorganization. During this time, both the luteal apoptosis and the reprogramming of gene expression in follicles are taking place. Studies have shown that feeding ruminants supplements containing one-carbon metabolites during the first 14 days of the estrous cycle significantly alters the ratio of SAM to SAH in the liver, and the concentration of folate cycle intermediates also changes in a dose-dependent manner (22), confirming that one-carbon metabolism is actively regulated during the estrous cycle. In ewes specifically, studies have shown that when pregnant animals are deficient in vitamin B12, methionine, and folate, their offspring develop epigenetic alterations. This demonstrates that the reproductive system is sensitive to methyl donor availability from this pathway (23). The glycine, serine, and threonine metabolism pathway also plays a role, as it is connected to the one-carbon cycle through serine's ability to donate methyl groups. This pathway was also significantly enriched in both the LP vs. LDP (RF = 0.062, *p* = 0.004) and LP vs. OES comparisons, further supporting the conclusion that one-carbon flux is broadly suppressed during luteolysis and then partially restored at estrus. Choline supplementation in pigs provides additional evidence. When pigs were given choline supplements, two important genes, LHR and CYP11A1, have been shown to upregulate expression in ovarian tissue and elevate levels of the fat-related molecule phosphatidylcholine in blood (10). This directly connect choline availability to two key abilities, how well the ovaries respond to reproductive hormones i.e., gonadotropin responsiveness and how well they produce steroid hormones i.e., steroidogenic capacity. Both of these mechanisms would be crucial for supporting the development of the preovulatory follicle during OES in our ewes.

### 4.2 Progressive acylcarnitine accumulation reflects carnitine-facilitated beta-oxidation toward estrus

A notable finding of the present dataset was the steady, step-by-step accumulation of short-chain acylcarnitines as the reproductive stages progressed from early LP to middle LDP to late OES. The two specific acylcarnitines, 3-hydroxybutyrylcarnitine [Car (4:0-O)] and acetyl carnitine [Car (2:0)], increased at each successive reproductive stage. Specifically, 3-hydroxybutyrylcarnitine rose from early LP stage to the middle LDP stage (FC = 1.284, *p* = 0.034, VIP = 1.670) and continued rising in the late-stage OES relative to the early-stage LP (FC = 1.775, *p* = 0.005, VIP = 2.580), while acetyl carnitine increased from the early LP stage to the late OES (FC = 1.341, *p* = 0.045, VIP = 1.844) and further increased from the middle LDP stage to the late OES (FC = 1.560, *p* = 0.010, VIP = 2.129). Considering the entire metabolic pathways, butanoate metabolism was significantly more active when comparing the middle LDP stage to the late OES stage (RF = 0.064, *p* = 0.003). Both acylcarnitine derivatives contributed to this enrichment, while propanoate metabolism was significantly more active when comparing the early stage to the late stage (RF = 0.048, *p* = 0.004). These results demonstrate that acylcarnitines accumulate progressively as the estrus approaches. These findings are consistent with increasing carnitine-facilitated β-oxidation activity as estrus approaches. However, direct evidence of enhanced β-oxidation flux would require experimental measurement of CPT1 activity and mitochondrial function, which were not assessed in the current work.

Acylcarnitines are formed by the conjugation of fatty acyl-CoA to carnitine by the enzyme called carnitine palmitoyltransferase-1 (CPT1), which is an essential step because it allows the fatty acids to be transported into the mitochondria for β-oxidation to produce energy (24). When β-oxidation (i.e., fatty acid oxidation) intensifies, short-chain acylcarnitines are released into the circulation when mitochondrial fatty acid import exceeds the capacity for complete oxidation. The observed increase in serum acylcarnitines, which began during the middle stage (LDP) and persisted through the late stage (OES), suggests that a metabolic shift toward greater reliance on fatty acid-derived energy may have been initiated during luteolysis and sustained as follicular development advanced. It should be noted, however, that serum acylcarnitine accumulation could also partially reflect incomplete mitochondrial oxidation rather than strictly enhanced β-oxidation activity. Functional validation of this interpretation remains to be addressed in future investigations. This is biologically plausible. The preovulatory follicle, the structure that releases the oocyte, is highly energy-demanding, requiring ATP for granulosa cell proliferation, expansion of the fluid-filled antrum cavity, and oocyte maturation. Research has shown that fatty acid β-oxidation plays a key supporting role in these energy-demanding processes (25).

A comprehensive review of carnitine biology in female reproductive physiology established that two forms of carnitine, L-carnitine and acetyl-L-carnitine, facilitate three key processes in oocytes: β-oxidation for energy production, membrane stabilization through phospholipid acetylation, and reduction of oxidative stress caused by reactive oxygen species. Each of these processes is critical for maintaining oocyte developmental competence during preovulatory maturation (11). Studies in reproductive medicine have shown that serum acylcarnitine concentrations strongly correlate with carnitine levels in ovarian follicular fluid, with short-chain acylcarnitine crossing the blood-follicle barrier most readily (26). These findings raise the possibility that the serum acylcarnitine elevations observed during the late stage (OES) in the present study may reflect increased fatty acid utilization within the follicular microenvironment. However, because direct measurement of intra-follicular acylcarnitine concentrations was not performed, this interpretation remains indirect. A study by Placidi et al. supported these findings, showing that acyl-carnitines have positive effects on mitochondrial activity in mouse oocytes under oxidative stress, specifically protecting the spindle apparatus and improving oocyte maturation rates (27). Collectively, the progressive buildup of acylcarnitines across LP, LDP, and OES, coupled with propanoate and butanoate pathway enrichment, points to a coordinated, phase-dependent metabolic shift toward greater fatty acid oxidation capacity as ewes near estrus. Direct functional studies are needed to determine whether this shift adequately supports the high-energy demands of preovulatory follicular development. This study only measured serum acylcarnitine levels and did not determine CPT1 activity or mitochondrial function-related indicators. Therefore, it cannot directly confirm the actual changes in fatty acid β-oxidation flux. Further validation through targeted indicators can elucidate its regulatory mechanism.

### 4.3 Consistent sphingosine downregulation reflects sphingolipid-mediated cell fate regulation during luteolysis

Sphingosine was the only metabolite that showed significant and consistent downregulation across all three pairwise comparisons. First, from the early LP to the middle LDP stage, sphingosine levels decreased to approximately 60% of their original value (FC = 0.604, *p* = 0.033, VIP = 1.712). Second, from early LP to the late OES stage, sphingosine levels decreased further to approximately 34% of their original (FC = 0.336, *p* = 0.006, VIP = 2.831), and finally, from the middle LDP to the OES stage, sphingosine levels decreased to approximately 56% of their LDP value (FC = 0.556, *p* = 0.009, VIP = 2.304). The sphingolipid signaling pathway was significantly enriched in two comparisons: LP vs. LDP (RF = 0.133, *p* = 0.005) and LP vs. OES comparisons (RF = 0.067, *p* = 0.036). Importantly, the apoptosis pathway that controls programmed cell death was enriched entirely through sphingosine in the early LP vs. late OES stage comparison (RF = 0.250, *p* = 0.010). This raises the possibility of a connection between sphingolipid dynamics and cell death signals during corpus luteum regression, but direct causal evidence remains to be established in future work.

To better understand these findings, it is useful to consider the ceramide/sphingosine-1-phosphate (S1P) rheostat, a lipid-based signaling system that helps determine whether ovarian cells survive or undergo apoptosis. In this system, two molecules, sphingosine and ceramide, promote cell growth arrest and cell death, whereas S1P exerts the opposite effect. S1P is generated when the enzym sphingosine kinase adds a phosphate group to sphingosine. S1P drives cellular survival, angiogenesis (growth of new blood vessels), and proliferation (28). These data show a progressive decline in serum sphingosine across the LP–LDP–OES stages, which is consistent with the hypothesis that sphingosine may be progressively converted to pro-survival S1P within ovarian cells, potentially acting as a counter-regulatory brake on PGF_2α_-induced luteal apoptosis. However, because neither S1P nor ceramide were directly measured here, this interpretation remains a hypothesis that requires direct experimental testing. The researchers used CIDR-PGF_2α_ treatment to control the reproductive cycle. Under this treatment, PGF_2α_ induces luteolysis by increasing the activity of two proteins called Bax and p53. These proteins activate a chain of reactions ultimately activating caspase-3 (29), leading to both functional and physical breakdown of the corpus luteum. A study by Parborell et al. in an animal model demonstrated that local administration of S1P effectively blocked PGF_2α_-induced luteolysis by suppressing the activity of caspase −2, −3, and −8, and by reducing the number of cells undergoing apoptosis in the corpus luteum (30). Published studies raise the possibility that the sphingosine-S1P axis contributes to luteal regression, even though this mechanism was not directly tested in the present study. A recent review of sphingolipid biology in follicle development established that granulosa cells produce S1P in response to follicle stimulating hormone. Once S1P is synthesized, it activates specific receptors on cells called S1PR2 and S1PR3, engaging two important signaling pathways: PI3K/Akt/mTOR and ERK/MAPK. These signaling pathways drive the follicle survival and growth (28). In the present study, the stepwise decline in serum sphingosine from LP through OES suggests that sphingosine-to-S1P conversion may progressively increase as the reproductive system transitions from luteal maintenance to follicular activation. If confirmed in future studies measuring S1P, ceramide, and sphingosine kinase activity directly, this would suggest that pro-survival sphingolipid signaling coordinates follicular development with ongoing luteal regression. This study only measured sphingosine levels and did not simultaneously measure key upstream and downstream molecules such as ceramides and S1P. The association between persistent downregulation of sphingosine and S1P-mediated cell survival signaling remains a reasonable hypothesis and awaits further verification in subsequent targeted experiments. Similar stage-dependent sphingolipid changes associated with ovarian function have been observed in other species. In yak serum, for example, follicular fluid metabolomic during estrus transitions revealed coordinated changes in lipid and amino acid metabolic pathways consistent with corpus luteum regression and follicular activation (31).

### 4.4 Glycerophospholipid remodeling supports follicular membrane expansion toward estrus

We found that glycerophospholipid metabolism was significantly enriched when comparing the middle LDP stage to the late OES stage (RF = 0.054, *p* = 0.006). This enrichment was driven by three specific metabolites: choline, glycerophosphocholine, and 1,2-dilinoleoyl-sn-glycero-3-phosphoethanolamine. All three of these metabolites were significantly elevated at OES compared to LDP. In addition, LPA(20:5) was significantly elevated in two comparisons: LP vs. LDP (FC = 1.976) and LP vs. OES (FC = 1.972). Another metabolite called PC (36:3) also increased in the same comparisons: LP vs. LDP (FC = 1.471) and LP vs. OES (FC = 1.378). These findings indicate that as ewes approach estrus, the glycerophospholipid pool in the body undergoes coordinated, stage-dependent remodeling. This remodeling process helps support the expansion of cell membranes in the developing follicle.

Glycerophospholipids are the main building blocks of cell membranes. As the folicle grows, it needs to produce more of these molecules to support several processes, e.g., granulosa cell expansion, antrum formation, and follicular fluid accumulation. The availability of glycerophospholipids has been identified as a key factor in determining whether a follicle is healthy and whether the oocyte is capable of developing properly. A two-stage metabolomics study in Communications Biology analyzed 452 follicular fluid samples and identified lysophosphatidylcholine as a significant predictor of follicular development and ovarian sensitivity to hormonal stimulation, highlighting the choline/glycerophospholipid pathway as a fundamental metabolic regulator of follicle maturation (32). Other Follicular fluid studies have also shown that multiple types of PC, a class of glycerophospholipid, change significantly depending on follicle size and age. These changes in PC are linked to the synthesis of another important mitochondrial lipid, cardiolipin, as well as to energy-producing pathways in the oocyte that rely on lipids (33). Beyond their role as structural building blocks, some glycerophospholipids, including LPA and LPC, actively participate in cell signaling within the ovary. Specifically, LPA helps regulate the activity of the enzyme COX-2 and the production of prostaglandins, hormone-like molecules involved in inflammation and reproduction. LPA also plays a role in helping granulosa cells survive and differentiate (34). These signaling functions are directly relevant to the changes that occur during luteolysis (corpus luteum breakdown), observed in our early LP and middle LDP comparisons. The coordinated nature of glycerophospholipid remodeling and choline recovery at the late OES stage is likely not coincidental but reflects a coordinated biological program. As choline becomes more available at the late OES stage, it is channeled directly into *de novo* phospholipid biosynthesis via the CDP-choline pathway (Kennedy pathway), producing new phospholipids for cell membrane synthesis. This increase in membrane synthesis drives the membrane expansion required for the preovulatory follicular growth. This connection between the choline metabolism and glycerophospholipid synthesis has been shown in previous research. Metabolomics studies of ovarian follicles have found that choline, lysophosphatidylcholine (LPC), and phosphatidylcholine (PC) do not act independently but function as an interconnected network of biosynthetic events involved in reproduction and cell membrane synthesis (32).

These findings are consistent with metabolomics analyses done in other ruminants, such as cows and goats, where glycerophospholipid metabolism consistently appears as one of the most strongly enriched pathways during ovarian transitions between reproductive states (35).

### 4.5 Other metabolic changes and integrated interpretation

Several additional metabolic findings provide important context for the overall biological picture. One metabolite, Pregnanediol 3-O-glucuronide, showed the highest fold change of any single metabolite in the dataset. It was elevated 4.6-fold in the late stage OES relative to the middle stage LDP (VIP = 2.491, *p* = 0.001). This metabolite is the major breakdown product of progesterone found in urine, produced when the liver breaks down progesterone via 5α-reductase pathway and conjugates it to glucuronic acid using the enzyme UDP-glucuronosyltransferase (36). Importantly, this sharp increase does not mean that progesterone production is high. In fact, high progesterone would be physiologically contradictory at estrus, as progesterone levels must be low for estrus to occur. Instead, the rise in this breakdown product documents the acceleration of hepatic progesterone clearance and its processing. This acceleration must occur for progestrone withrawal to proceed efficiently, allowing full estrus expression and the initiation of the LH surge, the hormone that triggers ovulation. Liver enzymes such as CYP2C and CYP3A, members of the cytochrome P450 family, are the primary mediators of progestrone breakdown. Their activity increases during luteolysis, which accelerates the removal of progestrone from the bloodstream (37). Therefore, the marked serum accumulation of the glucuronide conjugate in the blood at the late stage OES in the Kazak ewes studied confirms that this hepatic clearance process has been successfully completed.

Lactate levels were significantly elevated at the late OES stage relative to the middle LDP stage (FC = 1.522, VIP = 2.235, *p* < 0.001). In the same comparison, a carbon metabolism pathway was enriched through lactate and uracil. To understand the relevance of this finding, during follicle development, the growing follicle lacks a direct blood supply, creating a low-oxygen environment in which granulosa cells rely mainly on glycolysis to produce energy. These cells then export lactate and pyruvate to the oocyte as the primary metabolic substrate (38). Research has shown that lactate levels in the follicular fluid increase 2.5 times after the onset of the LH surge compared to mid-follicular levels, and this increase is closely linked to the size of the follicle and the amount of gonadotropin stimulation (39). A 2024 study in Human Reproduction also found that the ability of granulosa cells to perform glycolyis at the time of ovulation correlates directly with both oocyte quality and IVF success rates (25). Therefore, the elevation of serum lactate during the late OES stage in the present study supports the idea that granulosa cells increase their glycolysis during preovulatory follicular maturation, with extra lactate overflowing from the follicle into the blood circulation.

Among amino acid-related metabolites, two dipeptides, Glu-Arg and Ser-Ala, showed a clear rise-and-fall pattern. Both were significantly elevated in the middle stage LDP relative to the early stage LP (FC = 2.468 and 1.858), then both were markedly reduced at the late stage OES relative to the middle stage LDP (FC = 0.533 and 0.524). This pattern is consistent with the structural breakdown of luteal tissue during luteolysis, which releases amino acid dipeptides as byproducts. The rise in these dipeptides during the middle stage LDP reflects this breakdown. The subsequent fall during late stage OES reflects the body clearing away these byproducts and redistributing the amino acids as the reproductive system shifts from the luteal phase to the follicular phase. The process of breaking down proteins to release amino acids during luteolysis has been described as a conserved metabolic feature, meaning it is a common and stable characteristic of reproductive transitions in ruminant animals (35).

## 5 Conclusion

This is the first study to use UHPLC-MS/MS-based serum metabolomics to characterize the metabolic changes in Kazak ewes across three stages of hormone-induced estrus: LP (luteal, early stage), LDP (luteolysis, middle stage), and OES (estrus, late stage), identifying 213 significantly different metabolites when comparing LP to LDP, 119 when comparing LDP to OES, and 203 when comparing LP to OES. However, the use of HILIC chromatography in this study may limit the detection of less polar metabolites that could also play important roles in reproductive pathways. KEGG pathway enrichment analysis revealed coordinated, stage-dependent alterations in four key areas: one-carbon metabolism involving choline and folate, fatty acid β-oxidation involving carnitine, sphingolipid signaling involving sphingosine, and glycerophospholipid biosynthesis involving membrane building blocks. Five metabolites, choline, betaine aldehyde, 3-hydroxybutyrylcarnitine, acetyl carnitine, and sphingosine, emerged as the most biologically significant and stage-sensitive serum biomarkers in the hormone-induced estrus transition. These findings provide a quantitative metabolic reference for understanding reproductive physiology in Kazak ewes. They also lay the foundation for future comparative metabolomics studies in naturally cycling ewes, with the ultimate goal of developing nutritional strategies that target specific metabolites to induce estrus during the non-breeding season.

## Data Availability

The original contributions presented in the study are included in the article/Supplementary material. The metabolomics dataset has been deposited in MetaboLights (41) with the permanent accession number MTBLS15117, under CC0 1.0 license. Further inquiries can be directed to the corresponding author.

## References

[1] Duan Z Liu M Li J Hou J. Long-interval prostaglandin F2α combined with GnRH improves the estrus synchronization and reproductive performance of sheep during the breeding season. Animals. (2025) 15:336. doi: 10.3390/ani15030336 39943106 PMC11816233

[2] Mohan K Kumar N. Comparative evaluation of estrus synchronization protocols on reproductive performance and estrus behavior in Barbados Black Belly sheep. Vet World. (2023) 16:2244–9. doi: 10.14202/vetworld.2023.2244-2249 38152269 PMC10750750

[3] Zhang J Sun S Bai X Yang N Liu Y Wu X . Metabolomics analysis of the effect of GnRH on the pregnancy rate of ewes with estrus synchronization scheme based on progesterone. Front Vet Sci. (2024) 11:1442931. doi: 10.3389/fvets.2024.1442931 39055862 PMC11270128

[4] Nan Y Jiang B Qi X Ye C Xie M Zhao Z. Metabolomic and molecular mechanisms of glycerol supplementation in regulating the reproductive function of Kazakh ewes in the non-breeding season. Animals. (2025) 15:2291. doi: 10.3390/ani15152291 40805081 PMC12345530

[5] Fiehn O. Metabolomics — the link between genotypes and phenotypes. Plant Mol Biol. (2002) 48:155–71. doi: 10.1007/978-94-010-0448-0_11 11860207

[6] Johnson CH Ivanisevic J Siuzdak G. Metabolomics: beyond biomarkers and towards mechanisms. Nat Rev Mol Cell Biol. (2016) 17:451–9. doi: 10.1038/nrm.2016.25 26979502 PMC5729912

[7] Li Q Chao T Wang Y Xuan R Guo Y He P . Comparative metabolomics reveals serum metabolites changes in goats during different developmental stages. Sci Rep. (2024) 14:7291. doi: 10.1038/s41598-024-57803-7 38538719 PMC10973421

[8] Xu Q Wang C Wang L Feng R Guo Y Feng S . Correlation analysis of serum reproductive hormones and metabolites during multiple ovulation in sheep. BMC Vet Res. (2022) 18:290. doi: 10.1186/s12917-022-03387-1 35883090 PMC9317590

[9] Zhai Y Xia F Shi L Ma W Lv X Sun W . Early pregnancy markers in the serum of ewes identified via proteomic and metabolomic analyses. Int J Mol Sci. (2023) 24:14054. doi: 10.3390/ijms241814054 37762358 PMC10530974

[10] Zhan X Fletcher L Dingle S Baracuhy E Wang B Huber LA . Choline supplementation influences ovarian follicular development. Front Biosci. (2021) 26:1525–36. doi: 10.52586/5046 34994167

[11] Agarwal A Sengupta P Durairajanayagam D. Role of L-carnitine in female infertility. Reprod Biol Endocrinol. (2018) 16:5. doi: 10.1186/s12958-018-0323-4 29373970 PMC5785901

[12] Dunn WB Broadhurst D Begley P Zelena E Francis-McIntyre S Anderson N . Procedures for large-scale metabolic profiling of serum and plasma using gas chromatography and liquid chromatography coupled to mass spectrometry. Nat Protoc. (2011) 6:1060–83. doi: 10.1038/nprot.2011.335 21720319

[13] Zhou Z Luo M Zhang H Yin Y Cai Y Zhu ZJ. Metabolite annotation from knowns to unknowns through knowledge-guided multi-layer metabolic networking. Nat Commun. (2022) 13:6656. doi: 10.1038/s41467-022-34537-6 36333358 PMC9636193

[14] Sumner LW Amberg A Barrett D Beale MH Beger R Daykin CA . Proposed minimum reporting standards for chemical analysis chemical analysis working group (CAWG) metabolomics standards initiative (MSI). Metabolomics. (2007) 3:211–21. doi: 10.1007/s11306-007-0082-2 24039616 PMC3772505

[15] Saccenti E Hoefsloot HCJ Smilde AK Westerhuis JA Hendriks MMWB. Reflections on univariate and multivariate analysis of metabolomics data. Metabolomics. (2014) 10:361–74. doi: 10.1007/s11306-013-0598-6

[16] Kanehisa M Goto S. KEGG. kyoto encyclopedia of genes and genomes. Nucleic Acids Res. (2000) 28:27–30. doi: 10.1093/nar/28.1.27 10592173 PMC102409

[17] Kanehisa M Sato Y Kawashima M Furumichi M Tanabe M. KEGG as a reference resource for gene and protein annotation. Nucleic Acids Res. (2016) 44:D457–462. doi: 10.1093/nar/gkv1070 26476454 PMC4702792

[18] Goldansaz SA Guo AC Sajed T Steele MA Plastow GS Wishart DS. Livestock metabolomics and the livestock metabolome: a systematic review. PLoS ONE. (2017) 12:e0177675. doi: 10.1371/journal.pone.0177675 28531195 PMC5439675

[19] Sfakianoudis K Zikopoulos A Grigoriadis S Seretis N Maziotis E Anifandis G . The role of one-carbon metabolism and methyl donors in medically assisted reproduction: a narrative review of the literature. Int J Mol Sci. (2024) 25:4977. doi: 10.3390/ijms25094977 38732193 PMC11084717

[20] Goldansaz SA Markus S Plastow G Wishart DS. Predictive blood biomarkers of sheep pregnancy and litter size. Sci Rep. (2022) 12:10307. doi: 10.1038/s41598-022-14141-w 35725997 PMC9209467

[21] Safain KS Swanson KC Caton JS. The interplay of one-carbon metabolism, mitochondrial function, and developmental programming in ruminant livestock. J Dev Biol. (2026) 14:3. doi: 10.3390/jdb14010003 41562863 PMC12821658

[22] Crouse MS Freetly HC Lindholm-Perry AK Neville BW Oliver WT Lee RT . One-carbon metabolite supplementation to heifers for the first 14 days of the estrous cycle alters the plasma and hepatic one-carbon metabolite pool and methionine-folate cycle enzyme transcript abundance in a dose-dependent manner. J Anim Sci. (2023) 101:skac419. doi: 10.1093/jas/skac419 36566452 PMC9890446

[23] Syring JG Crouse MS Entzie YL King LE Hirchert MR Ward AK . One-carbon metabolite supplementation increases vitamin B12, folate, and methionine cycle metabolites in beef heifers and fetuses in an energy dependent manner at day 63 of gestation. J Anim Sci. (2024) 102:skae202. doi: 10.1093/jas/skae202 39028746 PMC11322739

[24] Montjean D Entezami F Lichtblau I Belloc S Gurgan T Menezo Y. Carnitine content in the follicular fluid and expression of the enzymes involved in beta oxidation in oocytes and cumulus cells. J Assist Reprod Genet. (2012) 29:1221–5. doi: 10.1007/s10815-012-9855-2 23054356 PMC3510375

[25] Morimoto A Rose RD Smith KM Dinh DT Umehara T Winstanley YE . Granulosa cell metabolism at ovulation correlates with oocyte competence and is disrupted by obesity and aging. Hum Reprod. (2024) 39:2053–66. doi: 10.1093/humrep/deae154 39013118 PMC11373349

[26] Várnagy A Bene J Sulyok E Kovács GL Bódis J Melegh B. Acylcarnitine esters profiling of serum and follicular fluid in patients undergoing *in vitro* fertilization. Reprod Biol Endocrinol. (2013) 11:67. doi: 10.1186/1477-7827-11-67 23866102 PMC3724743

[27] Placidi M Vergara T Casoli G Flati I Capece D Artini PG . Acyl-carnitines exert positive effects on mitochondrial activity under oxidative stress in mouse oocytes: a potential mechanism underlying carnitine efficacy on PCOS. Biomedicines. (2023) 11:2474. doi: 10.3390/biomedicines11092474 37760915 PMC10525604

[28] Tabasi R Ghasemian F Tavana S. Sphingolipids as key mediators in folliculogenesis and female fertility. Life Sci. (2025) 374:123697. doi: 10.1016/j.lfs.2025.123697 40348177

[29] Billhaq DH Lee S. The mechanisms of angiogenesis and apoptosis during the functional formation and regression of the corpus luteum in the ovarian reproductive endocrine system. Endocrines. (2025) 6:53. doi: 10.3390/endocrines6040053

[30] Hernandez F Peluffo MC Bas D Stouffer RL Tesone M. Local effects of the sphingosine 1-phosphate on prostaglandin F2alpha-induced luteolysis in the pregnant rat. Mol Reprod Dev. (2009) 76:1153–64. doi: 10.1002/mrd.21083 19645054 PMC4001830

[31] Feng F Huang C Luosang D Ma X La Y Wu X . Serum metabolomic analysis of synchronous estrus in yaks based on UPLC-Q-TOF MS technology. Animals. (2024) 14:1399. doi: 10.3390/ani14101399 38791618 PMC11117382

[32] Yang J Li Y Li S Zhang Y Feng R Huang R . Metabolic signatures in human follicular fluid identify lysophosphatidylcholine as a predictor of follicular development. Commun Biol. (2022) 5:763. doi: 10.1038/s42003-022-03710-4 35906399 PMC9334733

[33] Zhang X Wang T Song J Deng J Sun Z. Study on follicular fluid metabolomics components at different ages based on lipid metabolism. Reprod Biol Endocrinol. (2020) 18:42. doi: 10.1186/s12958-020-00599-8 32398082 PMC7216654

[34] Pan Y Pan C Zhang C. Unraveling the complexity of follicular fluid: insights into its composition, function, and clinical implications. J Ovarian Res. (2024) 17:237. doi: 10.1186/s13048-024-01551-9 39593094 PMC11590415

[35] Bai Y Song Y Zhang J Fu S Wu L Xia C . GC/MS and LC/MS based serum metabolomic analysis of dairy cows with ovarian inactivity. Front Vet Sci. (2021) 8:678388. doi: 10.3389/fvets.2021.678388 34490390 PMC8417594

[36] Yang G Ge S Singh R Basu S Shatzer K Zen M . Glucuronidation: driving factors and their impact on glucuronide disposition. Drug Metab Rev. (2017) 49:105–38. doi: 10.1080/03602532.2017.1293682 28266877 PMC7660525

[37] Wiltbank MC Gümen A Sartori R. Physiological classification of anovulatory conditions in cattle. Theriogenology. (2002) 57:21–52. doi: 10.1016/S0093-691X(01)00656-2 11775971

[38] Han Y Wang X Chen Z Liu T Pan B Hu S . Lactate promotes hypoxic granulosa cells' autophagy by activating the HIF-1α/BNIP3/Beclin-1 signaling axis. Genes. (2025) 16:14. doi: 10.3390/genes16010014 39858561 PMC11765430

[39] Leese HJ Barton AM. Pyruvate and glucose uptake by mouse ova and preimplantation embryos. J Reprod Fertil. (1984) 72:9–13. doi: 10.1530/jrf.0.0720009 6540809

[40] Lunsin R Sokantat D Silvestre T Neto HRL Koh TJ Sun F . Metabolic status, reproductive, and productive performances of transition dairy cows as affected by dietary rumen-protected choline supplementation. J Adv Vet Anim Res. (2024) 11:754–61. doi: 10.5455/javar.2024.k827 39605753 PMC11590597

[41] Yurekten O Payne T Tejera N Amaladoss FX Martin C Williams M . MetaboLights: open data repository for metabolomics. Nucleic Acids Res. (2024) 5:D640–D646. doi: 10.1093/nar/gkad1045 37971328 PMC10767962

